# Different Gene Expression Signatures in Children and Adults with Celiac Disease

**DOI:** 10.1371/journal.pone.0146276

**Published:** 2016-02-09

**Authors:** V. Pascual, L. M. Medrano, N. López-Palacios, A. Bodas, B. Dema, M. Fernández-Arquero, B. González-Pérez, I. Salazar, C. Núñez

**Affiliations:** 1 Servicio de Inmunología Clínica, Instituto de Investigación Sanitaria del Hospital Clínico San Carlos (IdISSC), Madrid, Spain; 2 Servicio de Pediatría, Instituto de Investigación Sanitaria del Hospital Clínico San Carlos (IdISSC), Madrid, Spain; 3 Servicio de Aparato Digestivo, Instituto de Investigación Sanitaria del Hospital Clínico San Carlos (IdISSC), Madrid, Spain; 4 Departamento de Estadística e Investigación Operativa I, Facultad de Matemáticas, Universidad Complutense de Madrid, Madrid, Spain; 5 Departamento de Producción Animal, Facultad de Veterinaria, Universidad Complutense de Madrid, Madrid, Spain; Baylor College of Medicine, UNITED STATES

## Abstract

Celiac disease (CD) is developed after gluten ingestion in genetically susceptible individuals. It can appear at any time in life, but some differences are commonly observed between individuals with onset early in life or in adulthood. We aimed to investigate the molecular basis underlying those differences. We collected 19 duodenal biopsies of children and adults with CD and compared the expression of 38 selected genes between each other and with the observed in 13 non-CD controls matched by age. A Bayesian methodology was used to analyze the differences of gene expression between groups. We found seven genes with a similarly altered expression in children and adults with CD when compared to controls (*C2orf74*, *CCR6*, *FASLG*, *JAK2*, *IL23A*, *TAGAP* and *UBE2L3*). Differences were observed in 13 genes: six genes being altered only in adults (*IL1RL1*, *CD28*, *STAT3*, *TMEM187*, *VAMP3* and *ZFP36L1*) and two only in children (*TNFSF18* and *ICOSLG*); and four genes showing a significantly higher alteration in adults (*CCR4*, *IL6*, *IL18RAP* and *PLEK*) and one in children (*C1orf106*). This is the first extensive study comparing gene expression in children and adults with CD. Differences in the expression level of several genes were found between groups, being notorious the higher alteration observed in adults. Further research is needed to evaluate the possible genetic influence underlying these changes and the specific functional consequences of the reported differences.

## Introduction

Celiac disease (CD) is an immune-mediated systemic disease characterized by a chronic small-bowel enteropathy triggered by dietary gluten ingestion in genetically susceptible individuals. The knowledge of CD has highly increased in the last decades. From the first considerations as a rare disease mostly affecting children, it has evolved to a common disease that can appear at any age[1]. Nowadays, it is recognized that there may exist an adult-onset of CD, although marked differences are commonly observed between children and adults presenting the disease. The classical clinical presentation, characterized by gastrointestinal symptoms with malabsorption, is more common in young children. Adults commonly show more subtle clinical manifestations, dominated by nutritional deficiencies or unspecific extraintestinal symptoms or even show subclinical disease[2]. A much higher frequency of other autoimmune diseases is also observed in adult patients. Differences in other important features, such as CD-specific antibodies, HLA-associated genetics or severity of the mucosal lesion can be also found between these two groups[3, 4]. Recently, age-related differences in CD have been extensively reviewed [5, 6].

CD etiology is not completely known, but dietary gluten and a specific genetic background are two mandatory factors for CD development. This disease shows a strong genetic influence, but despite the huge advances in the knowledge of the genetic architecture of CD, this is far to be completely understood. The main genetic susceptibility factors lie on the HLA region and encode the HLA-DQ2/DQ8 heterodimers, but additional multiple low-risk genetic variants located within diverse loci across the genome have been described[7]. However, these variants are mere markers of susceptibility and their translation to causal genes is still a major challenge of research in CD. Different approaches have been followed to deal with that issue and nowadays it is known that most of the causal variants have a regulatory role and modulate gene expression[8, 9].

On the other hand, despite the huge advances prompted by large-scale genetic studies, the described genetic variants explain around 48% of CD heritability[10], leaving a considerable fraction to be determined. These studies require high numbers of individuals to reach statistical significance and therefore they typically consider all patients as a whole. This practice allows the identification of genetic variants affecting to the entire group or to the majority of patients, but may mask susceptibility variants of specific subgroups. It neither allows us to distinguish putative differences in the risk level conferred by each susceptibility factor to individual subgroups.

Gene expression studies have demonstrated to be a useful approach to refine the causal gene/s in the described risk loci and also to provide evidence of the implication of several functional pathways in complex diseases, such as CD[11–13]. This kind of studies does not need the high sample sizes mandatory in GWAS and may be helpful to address the comparative study between pediatric and adult patients with CD. Based on this, we selected two groups of CD patients stratified according to the age at diagnosis and analyzed the expression of several genes selected by being located in loci associated to CD susceptibility or by being involved in the Th17 immune response, which has been associated to the pathogenesis of several immune-mediated diseases[14, 15] including CD[13, 16–18].

## Materials and Methods

### Subjects

We studied 19 CD patients: 13 children and 6 adults; and 13 controls: 7 children and 5 adults. CD was diagnosed according to the European Society for Paediatric Gastroenterology Hepatology and Nutrition (ESPGHAN) criteria[19] in children (all the samples were collected before January 2012) and according to the American Gastroenterological Association (AGA) recommendations[20] in adults. All CD patients showed CD specific antibodies and villous atrophy (Marsh 3a-3c) and carried the alleles-encoding HLA-DQ2. Subjects with normal histology and without immune- related diseases were used as controls. The median age of the studied subjects was 5.6 ± 0.6 for CD children, 8.1 ± 2.2 for control children, 38.3 ± 3.4 for CD adults and 50.6 ± 10.9 for control adults.

A written informed consent was obtained from the adult participants and from the parents of the children participants. This study was approved by the Ethics Committee of the Hospital Clínico San Carlos.

### Tissue samples, RNA isolation and gene expression

Duodenal biopsies were obtained during routine diagnostic endoscopy. Tissue samples were immediately immersed in RNA later, kept at 4°C 24–48 hours and subsequently stored at -70°C until use. RNA was obtained using the RNeasy Mini Kit (Qiagen, Westburg, Leuden, The Netherlands). Quantity and purity of RNA was determined by spectrophotometry (Nanodrop, Thermo Scientific NanoDrop Products, Wilmington, DE, USA). RNA integrity was assessed by a RNA quality assay (RQ1 and RQ2) (BioRad Laboratories, Hercules, CA, USA). cDNA was obtained by RT-PCR reaction (High Capacity RNA-to-cDNA Master Mix, Applied Biosystems, Foster City, CA, USA).

The expression of 48 genes was measured by quantitative PCR (qPCR) using Custom TaqMan Array Cards (format 384-well microfluidic card, Applied Biosystems, Foster City, CA, USA) (S1 Table). Thirty-one of these genes were located in 18 loci associated to CD risk and 13 genes were functionally related to the Th17 immune response, two genes could be included in both groups. For one of the analyzed genes, *IL1RL1*, two different probes were included in order to discriminate the isoform encoding the membrane-bound protein and the one encoding the soluble protein. Three commercially available housekeeping genes were included (*RPLP0*, *HPRT1* and *18s*), but none of them could be used as an endogenous reference gene in our sample. We evaluated our target genes to look for reference genes by using two algorithms: geNorm version 3.5 [21] and NormFinder version 0.953[22]. *PUS10* and *GLB1* genes showed high stability and could be used as endogenous reference genes. The mRNA expression level of each gene was calculated according to delta-Ct (ΔC_t_ = ΔC_t target_ − ΔC_t endogenous_ (), where lower ΔCTs represent more copy numbers of amplified mRNA. In graphs, the expression levels were presented using the comparative Ct method (2^-ΔΔCt^).

### Statistical analysis

We applied a previously described Bayesian methodology [23] to analyze the possible differences in the gene expression level of 38 genes between CD children and non-CD control children and between CD adults and non-CD control adults. We also evaluated differences between children and adults with CD, and between non-CD control children and adults. In each group, we tested simultaneously the 38 genes, considering the multiple testing problem given by

$$H_{0i}:\;\mu_{i}=0H\;\mathrm{vs}.\;H_{1i}:\;\mu_{i}\neq 0,\;i=1,\ldots ,38H,$$

and the statistic Ti, difference of means between the two compared groups.

The goal of this methodology is to obtain the posterior probability of each null hypothesis, given the observed value of all statistics, Pr(μ_i_ = 0|t_1_, …, t_38_), or equivalently the posterior probability of each alternative hypothesis, Pr(μ_i_ ≠ 0|t_1_, …, t_38_) and then decide accordingly.

To determine which null hypotheses will be accepted and which ones will be rejected, on the basis of the posterior probability, we considered a widely used criterion from a Bayesian decision theory point of view. This criterion is to apply the Bayes rule for the loss function described in Gomez-Villegas et al. [24] with equal cost for a false positive and a false negative for all genes. Thereby, each null hypothesis will be rejected

$$\text{if}\;\text{Pr}(\mu_{\text{i}}=0\left|{\text{t}}_{1},\ldots ,{\text{t}}_{38})\leq 0.5\text{Pr}(,\;\text{or equivalently if}\;{\text{Pr}(\mu }_{\text{i}}\neq 0\right|{\text{t}}_{1},\ldots ,{\text{t}}_{38})>0.5.$$

## Results

**Fig 1:**
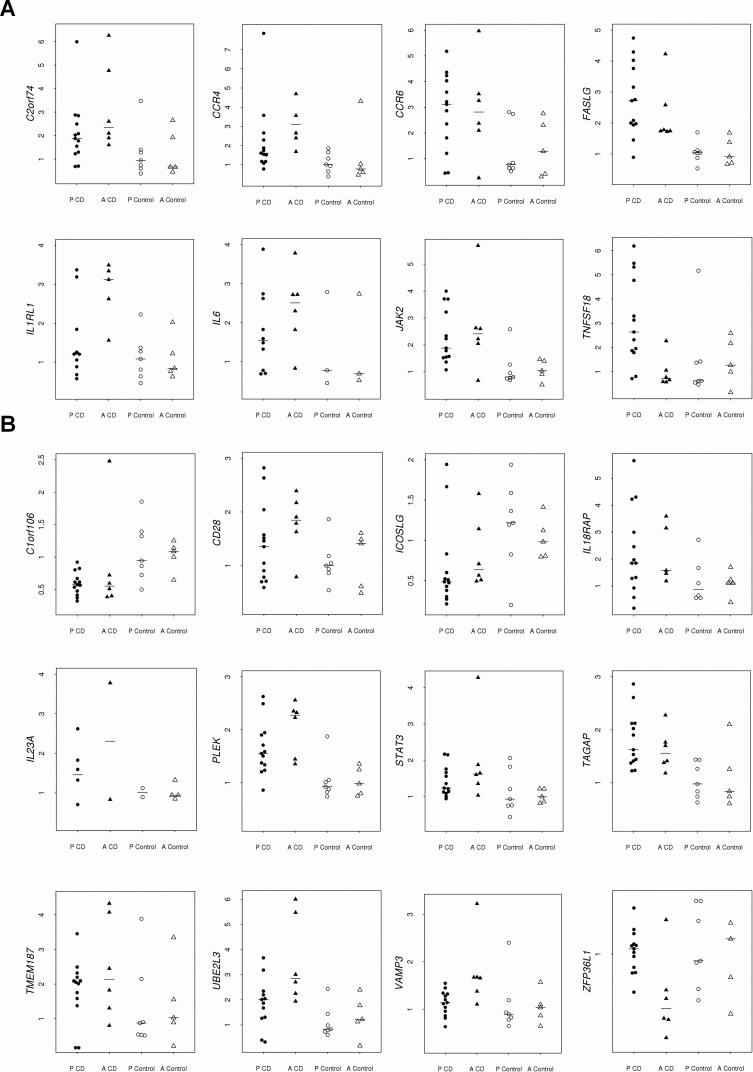
Relative expression in intestinal biopsies of children and adults with CD and non-CD control children and adults of the genes altered in at least one group of patients with A) high significance and B) low significance. Pediatric patients and controls are represented by black and white circles, respectively; and adult patients and controls are represented by black and white triangles, respectively.

Two of the selected genes, *ADAD1* and *OLIG3*, were under the limit of detection in all the samples. *IL17F*, *IL21* and *IL22* were discarded from the statistical analysis because they were not detected in the majority of the samples. However, expression of *IL21* was more frequently observed in patients, since it was detected in 8 out of the 13 CD children vs. 2 out of the 7 non-CD control children and in 3 out of the 6 CD adults vs. 0 out of the 5 non-CD control adults. Despite the reduced statistical power, low but non-significant results were obtained after performing these comparisons by using the Fisher´s exact test (p = 0.17 in children and p = 0.12 in adults). Excluding the previously mentioned five genes and the reference genes, the expression level of 38 genes was compared by a Bayesian procedure between CD patients and controls after being stratified according to the age at diagnosis. The inspection of the results allowed us to distinguish two groups of significant genes: those with a clearly altered expression in CD and those with a putative role in the disease which warrants further research (Table 1, Fig 1).

**Table 1:** Posterior probabilities obtained from the comparison between CD patients and controls stratified according a childhood- or adult-onset and between children and adults with CD.

Gene	CD vs. Controls		CD children vs. CD adults
	Children	Adults	
*C1orf106*	0.57	0.51	0.52
*C2orf74*	0.58	0.73	-
*CCR4*	0.58	0.79	0.72
*CCR6*	0.68	0.62	-
*CD28*	-	0.55	-
*FASLG*	0.72	0.63	-
*ICOSLG*	0.59	-	0.57
*IL18RAP*	0.54	0.60	0.51
*IL1RL1* (Hs01073297_m1)	-	0.72	0.70
*IL23A*	0.70	0.69	-
*IL6*	0.50	0.63	0.91
*JAK2*	0.65	0.65	-
*PLEK*	0.52	0.61	0.51
*STAT3*	-	0.56	-
*TAGAP*	0.56	0.52	-
*TMEM187*	-	0.62	-
*TNFSF18*	0.73	-	0.54
*UBE2L3*	0.52	0.77	-
*VAMP3*	-	0.55	-
*ZFP36L1*	-	0.57	-

The group of genes with a clear significantly altered expression in CD (Fig 1A) comprises four genes which are similarly up-regulated in children and adults with CD: *C2orf74*, *CCR6*, *FASLG* and *JAK2*; one gene uniquely up-regulated in CD children: *TNFSF18*; one gene uniquely up-regulated in CD adults: *IL1RL1* (the isoform encoding the soluble protein); and two genes most likely altered in both groups of patients, but with a higher effect in adults: *CCR4* and *IL6*. A highly significant value for *IL6* was only observed when considering the adult group, but it must be highlighted that this gene was detected in few samples, which were the ones used for statistical calculations. However, *IL6* was detected in 10 out of the 13 CD children vs.3 out of the 7 non-CD control children; which suggests that it is over-expressed in children with CD, also supported by the observed posterior probability between these two groups of 0.50.

A second group of genes showed lower significant differences between groups and a lower effect (Fig 1B). In this subset, six genes were altered in CD children and adults: *IL18RAP*, *IL23A*, *PLEK*, *TAGAP*, *UBE2L3* and *C1orf106*. The last gene was the only one down-regulated in CD patients and showed a higher effect in children. *IL18RAP* and *PLEK* showed higher up-regulation in CD adults. We also found one gene uniquely down-regulated in CD children: *ICOSLG;* and five genes up-regulated in CD adults: *CD28*, *STAT3*, *TMEM187*, *VAMP3* and *ZFP36L1*. The *IL18R1* gene showed a very low effect only in pediatric CD (p = 0.51).

Of note, some of the studied genes, such as *C1orf106*, *IL6* and *TNFSF18* showed differences in the expression level between children and adults in both patients and controls, which makes mandatory to analyze both groups separately unless patients and controls are matched by age.

No correlation was found between the expression level of any of the studied genes and the age of the subjects.

## Discussion

A common feature of CD is the presence of enteropathy triggered by gluten ingestion. This can be observed in individuals of any age, which despite this similarity, differ in several other characteristics, mainly depending on their childhood- or adult-onset of CD. In this work, we have investigated whether those differences may have a genetic or molecular basis, since this represents a question still unanswered. We selected children and adults with CD and compared the expression of several selected genes between each other and with the expression levels observed in non-CD controls matched by age. Notably, important differences were observed between these two groups of patients. We applied a previously described Bayesian methodology [23] and successfully used for multiple testing [24]. The frequentist approaches commonly used to deal with this problem are very conservative. They detect a low number of significant differences between groups and show a low number of false positives, but the number of false negative results is high. The procedure presently used detects more differentially expressed genes with a lower number of false negatives, resulting in a more powerful test, while the number of false positives remains at acceptable levels.

We found that 20 out of the 38 studied genes were differentially regulated in CD. Out of the 12 genes which showed a low significance and a low effect, six of them have been previously reported: *C1orf106*, *CD28*, *IL18RAP*, *PLEK*, *TAGAP* and *ZFP36L1*[9, 25–27].In all cases, concordance relative to down- or up-regulation was found between ours and previously published results. An increased expression of *IL21* and *FASLG* in active CD has been also described [25, 27], and *IL6* and *IL23A* have been found at higher although non-significant levels in CD patients [16]. In addition, several genes with similar expression in active CD and controls in our study have neither been observed as differentially regulated in previous works [27, 28]. We consider that these observations support the validity of the Bayesian methodology to deal with this problem, even in the cases of low significance.

The *SH2B3* gene, previously described as up-regulated in CD[25, 26, 29], showed a similar expression in our patients and controls. In contrast, we found several genes up- or down-regulated in CD which have been not previously reported. The detection of a higher number of significant results is not surprising considering the methodology used. Moreover, some of the discrepancies correspond to genes that we observed only altered in adults, while they have been previously studied in children. This statistical method allows us to work with small sample sizes, which is beneficial since sometimes it is complicated to collect a high number of samples. The possibility remains that our studied groups are not truly representative of the entire group of children and adults with CD. This could explain the discrepancy observed regarding to *SH2B3*, although the high concordance with the literature supports our results.

Undoubtedly, the most important finding of our study is that some genes are differentially regulated in children and adults with the disease. In particular, *TNFSF18* and *ICOSLG* were only differentially expressed in CD children and *IL1RL1*, *CD28*, *STAT3*, *TMEM187*, *VAMP3* and *ZFP36L1* were uniquely altered in adults with the disease. However, the last five genes did not show significant differences between children and adult patients, which could be a consequence of either false positive results in the group of adults or lower effects in children that hampered reaching a significant result. Since *CD28* and *ZFP36L1* have been described as up- and down-regulated, respectively, in CD children [27], the last possibility seems more plausible. This result would be in concordance with our observation in *CCR4*, *IL6*, *IL18RAP* and *PLEK*, which showed a higher deregulation in adult patients.

At our knowledge, this is the first work that reports differences in gene expression depending on the age of onset. Very recently, a different pattern of expression of some microRNAs, which can have a regulatory role on gene expression, has been described between children and adults with CD[30]. These observations constitute promising results that would help to explain why some individuals develop the disease early in life or later in adulthood and they could contribute to understand some of the differences observed between these two groups of patients, albeit further validation is warranted. It is very interesting that adults seem to show higher alteration in the expression of a considerable number of genes. It could be speculated that small changes contribute to develop CD in children, but deeper alterations are required in adults, which would explain why the disease is less frequent in this group.

Serum markers and histological alterations in biopsy specimens are usually milder in adults, which is associated with a longer delay in their diagnosis. For this study, we have chosen patients with positive serology and HLA-DQ2 and showing intestinal atrophy. The possibility remains that bigger differences emerge when considering adults with milder characteristics.

In addition, the methodology presently used is very sensitive and allows the detection of genes with low effect. The clinical implications of these findings must be further investigated, although it has been previously suggested that small shifts in the expression of susceptibility genes may have high impact if several genes involved in the same pathway are simultaneously affected [9]. Following this idea, it is notorious that we found seven (*CCR6*, *JAK2*, *IL21*, *IL6*, *IL23A*, *STAT3* and *VAMP3*) out of the eleven analyzed Th17-functionally related genes altered in CD. This suggests the involvement of a deregulated Th17 immune response in CD pathogenesis, with a higher contribution to adult disease. Of note, higher IL-21 but not IL-17A production was previously observed in pediatric CD patients, however, a subgroup of adult CD patients showed a higher production of IL-17A, which was postulated to be related to a higher contact with microbe-associated molecular patterns in CD adults[31]. To deepen into the possible functional differences between children and adults with the disease, we performed pathway analysis by using the STRING database (‘Search Tool for the Retrieval of Interacting Genes/Proteins’)[32]. Differences between these two groups of patients were observed in several GO biological process terms: positive regulation of response to wounding, positive regulation of inflammatory response and positive regulation of response to external stimulus, which seem to have special relevance in adults with CD. It is also notorious that the two genes showing an altered expression only in children, *TNFSF18* and *ICOSLG*, are grouped in the same gene network because they act in shared signaling pathways. These results could be indicating that children and adults respond differently in certain circumstances, which could contribute to the observed differences. Further research focusing on these processes could offer interesting results.

It must be underlined that the genes included in this work constitute only a reduced subset of potential genes related with CD, new studies including additional genes will probably reveal new differences between these two groups of patients which may impact on different fields. As a matter of fact, it could be relevant in order to select the group of individuals with high probability of responding properly to the currently being developed new therapies for CD. These therapies are based on different strategies and it remains the possibility that children and adults could differentially respond to some of them.

Since some differences in the genes located in risk loci were also observed, it could be advisable to reanalyze GWAS data according to the age of onset of the patients. This could facilitate the identification of new susceptibility variants and could contribute to reduce the fraction of heritability still unknown, the so called "missing heritability". In addition, it could help to find new genetic markers with diagnostic purposes, especially in the group of adults, which have been scarcely represented in GWAS studies and commonly show a difficult diagnosis.

## Supporting Information

S1 Table List of the studied genes with the corresponding TaqMan assays used. (DOCX)
