# Supplementary material for: Different Gene Expression Signatures in Children and Adults with Celiac Disease
Source: PLoS One. 2016 Feb 9;11(2):e0146276. doi: 10.1371/journal.pone.0146276 (PMC4747499; doi:10.1371/journal.pone.0146276)
Supplement: S1 Table — (DOCX) [file pone.0146276.s001.docx]

**S1. Table** List of the studied genes with the corresponding TaqMan assays used.

| **Gene** | **Taqman assay** | **Group of study** |
| --- | --- | --- |
| *18s* | Hs99999901_s1 | Housekeeping gene |
| *BACH2* | Hs00222364_m1 | Risk loci (6q15) |
| *C1orf106* | Hs01009089_m1 | Risk loci (1q32.1) |
| *C2orf74* | Hs01376007_m1 | Risk loci (2p16.1) |
| *CCR4* | Hs99999919_m1 | Risk loci (3p22.3) |
| *CCR6* | Hs01890706_s1 | Th17 pathway |
| *CD28* | Hs01007422_m1 | Risk loci (2q33.2) |
| *FASLG* | Hs00181225_m1 | Risk loci (1q24.3) |
| *FBXO48* | Hs01043600_m1 | Risk loci (2p13.3) |
| *GLB1* | Hs01035168_m1 | Risk loci (3p22.3) |
| *HCFC1* | Hs00232039_m1 | Risk loci (Xq28) |
| *HPRT1* | Hs99999909_m1 | Housekeeping gene |
| *ICOSLG* | Hs00323621_m1 | Risk loci (21q22.3) |
| *IL17F* | Hs00369400_m1 | Th17 pathway |
| *IL18R1* | Hs00977691_m1 | Risk loci (2q12.1) |
| *IL18RAP* | Hs00977695_m1 | Risk loci (2q12.1) |
| *IL1RL1* | Hs01073297_m1* | Risk loci (2q12.1) |
| *IL1RL1* | Hs01073300_m1# | Risk loci (2q12.1) |
| *IL2* | Hs00174114_m1 | Risk loci (4q27) |
| *IL21* | Hs00222327_m1 | Risk loci (4q27) and Th17 pathway |
| *IL22* | Hs01574154_m1 | Risk loci (12q15) and Th17 pathway |
| *IL23A* | Hs00372324_m1 | Th17 pathway |
| *IL23R* | Hs00332759_m1 | Th17 pathway |
| *IL6* | Hs00985639_m1 | Th17 pathway |
| *IRAK1* | Hs01018347_m1 | Risk loci (Xq28) |
| *JAK2* | Hs00234567_m1 | Th17 pathway |
| *OLIG3* | Hs00703087_s1 | Risk loci (6q23) |
| *PLEK* | Hs00950975_m1 | Risk loci (2p14) |
| *PTPN2* | Hs00959896_m1 | Risk loci (18p11.21) |
| *PUS10* | Hs00328708_m1 | Risk loci (2p16.1) |
| *REL* | Hs00968440_m1 | Risk loci (2p16.1) |
| *RHBDD3* | Hs00202161_m1 | Th17 pathway |
| *RPLP0* | Hs99999902_m1 | Housekeeping gene |
| *SH2B3* | Hs00193878_m1 | Risk loci (12q24.12) |
| *SMAD3* | Hs00969210_m1 | Th17 pathway |
| *STAT3* | Hs00374280_m1 | Th17 pathway |
| *TAGAP* | Hs01097874_m1 | Risk loci (6q25.3) |
| *TEC* | Hs00938888_m1 | Risk loci (4p11) |
| *TMEM187* | Hs01920894_s1 | Risk loci (Xq28) |
| *TNFAIP3* | Hs00234713_m1 | Risk loci (6q23.3) |
| *TNFSF18* | Hs00183225_m1 | Risk loci (1q24.3) |
| *TYK2* | Hs01105959_g1 | Th17 pathway |
| *UBE2L3* | Hs01062161_g1 | Risk loci (22q11.21) |
| *VAMP3* | Hs00922166_m1 | Th17 pathway |
| *YDJC* | Hs00419215_g1 | Risk loci (22q11.21) |
| *ZFP36L1* | Hs00245183_m1 | Risk loci (14q24.1) |
| *ZMIZ1* | Hs00393480_m1 | Risk loci (10q22.3) |

*Isoform encoding the soluble protein; # isoform encoding the soluble protein and the membrane-bound protein.
